# Optimizing cardio‐respiratory motion management in stereotactic arrhythmia radioablation through mid‐position planning target volume

**DOI:** 10.1002/acm2.70170

**Published:** 2025-07-14

**Authors:** Adèle Gabillaud, Louis Rigal, Raphaël Martins, Renaud De Crevoisier, Juan Cisneros, Loïg Duvergé, Mathieu Lederlin, Nolwenn Delaby, Karim Benali, Antoine Simon, Julien Bellec

**Affiliations:** ^1^ Department of Medical Physics CLCC Eugène Marquis Rennes France; ^2^ Univ Rennes, CHU Rennes, CLCC Eugène Marquis Inserm Rennes France; ^3^ Department of Radiation Oncology CLCC Eugène Marquis Rennes France; ^4^ Department of Cardiology Saint‐Etienne University Hospital Saint‐Priest‐En‐Jarez France

**Keywords:** cardiac radioablation, mid‐position, motion management, stereotactic arrhythmia radioablation

## Abstract

**Purpose/objective:**

This study proposes an implementation of the mid‐position (MidP) approach to compensate for cardio‐respiratory motions in the context of Stereotactic Arrhythmia Radioablation (STAR) and evaluates its benefits compared to an internal target volume (ITV) approach.

**Materials and methods:**

Fifteen patients who underwent STAR for refractory ventricular tachycardia in our institution were included in this retrospective planning study. For each patient, a cardiac‐gated four‐dimensional computed tomography (4D‐CT_card_) scan and a respiratory‐gated four‐dimensional computed tomography (4D‐CT_resp)_ were acquired. All patients were treated using a volumetric modulated arc therapy technique using an in‐treatment Cone‐Beam CT (CBCT) image guidance. The MidP approach was implemented to compensate for uncertainties, including cardio‐respiratory motions characterized using the 4D‐CT_card_ and 4D‐CT_resp_ scans, and the inter‐fraction motions measured using the CBCT scans. For comparison purposes, the ITV approach was also implemented. Both approaches were compared in terms of planning target volume (PTV) volumes, doses to organs‐at‐risk, and clinical target volume (CTV) doses, assessed using a 4D modeling method that estimates the accumulated dose.

**Results:**

Compared with the ITV method, the MidP approach resulted in a mean [min‐max] relative PTV volume reduction of 30% [19%, 48%] (*p* < 0.001, Wilcoxon signed rank test). The mean [min‐max] D95% CTV coverage was 105% [101%–114%] and 107% [101%–117%] of the prescription dose for MidP and ITV‐based plans, respectively. The median dose to the whole heart was significantly lower with MidP‐based plans with a mean difference of −0.5 Gy (*p* = 0.0084). The near‐maximum dose (D1%) delivered to left coronary arteries, aorta, and stomach was systematically lower with the MidP‐based plans.

**Conclusion:**

Compared to ITV based approach, the use of MidP strategy for treatment planning of STAR leads to significantly smaller PTV and lower surrounding OAR doses while still achieving a clinically acceptable CTV coverage.

## 1 INTRODUCTION

Ventricular Tachycardia (VT) is an arrhythmia, often caused by myocardial scar, causing an elevation in heart rate and, in severe cases, leading to the patient's death.^1^ The standard medical approach for managing VT includes the administration of anti‐arrhythmic medications, the implantation of a cardioverter–defibrillator (ICD), and a minimally invasive procedure called catheter radiofrequency ablation (CRF)^1^ stereotactic arrhythmia radioablation (STAR), also known as cardiac radioablation (CR) or cardiac stereotactic body radiotherapy (cardiac SBRT)^2^ has recently emerged to treat VT refractory to these conventional treatment techniques.^1^, ^2^, ^3^ It consists in delivering a dose of 20 to 25 Gy^3^, ^4^ in a single fraction to a clinical target volume (CTV), which is defined as the arrythmogenic region within the heart muscle responsible for the VT.

One of the most important issues in STAR is the management of target motions induced by both respiration and heartbeats.^5^ Prusator et al.^6^ reported a range of cardiac target motion amplitudes of [1–5] mm in left/right, [3–7] mm in anterior/posterior, and [1–9] mm in cranio/caudal (CC) directions, as well as respiration‐induced target motion amplitudes of [2–7] mm in left/right, [2–5] mm in anterior/posterior, and [2–8] mm in CC directions. Several motion management techniques, known as active or passive strategies, may be used for the management of cardiac and/or respiratory motions.^6^, ^7^ Active techniques, such as tracking and gating, directly adjust the treatment delivery in real‐time to account for the tumor's actual movement. On the other hand, passive techniques aim to mitigate motion effects prior to the treatment by incorporating a specific margin into the planning target volume (PTV). In clinical practice, when implementing STAR on conventional C‐arm Linacs, passive techniques are generally used.^3^, ^8^, ^9^ The most used passive motion management technique for STAR is the internal target volume (ITV) strategy^9^ according to which the PTV is designed to encompass the whole cardio‐respiratory target motion. Cardio‐respiration motions are evaluated using cardiac‐gated and/or respiration‐gated four‐dimensional computed‐tomography (4D‐CT) scans.^7^, ^9^ An additional generic margin is usually added to account for other treatment uncertainties (e.g., localization accuracy, intra‐fraction motions).^9^, ^10^ The main advantage of using an ITV‐based strategy for STAR is to ensure excellent target coverage.^11^ However, this strategy increases PTV volumes^12^ and therefore the exposure of surrounding healthy tissues. In a previous study, we have reported an increase of target volumes ranging from 76% to 249% compared to the CTV when generating an ITV‐based PTV.^7^

An alternative passive motion management technique is the mid‐position (MidP) strategy. PTV margins are then calculated around the time‐weighted mean target position by using a probabilistic margin recipe^13^ considering target motions as a random source of error among others. The MidP approach has been widely described and evaluated in the literature for management of respiratory motions for oncological indications including lung, esophagus, and pancreatic cases.^10^, ^11^, ^14^, ^15^, ^16^ The advantage of using MidP strategy, especially for targets presenting large respiratory motion amplitude, is to reduce PTV volumes, compared with the ITV, while still ensuring an acceptable CTV coverage.^10^, ^11^

To the best of our knowledge, the MidP concept has not been yet implemented and evaluated for STAR. The specificity of STAR lies in the fact that the target moves with both the respiration and heartbeats, so the implementation of the MidP strategy should account for both components. Moreover, evaluating these approaches in the context of these complex dynamics remains an open question, since the impact of the combination of motions, in terms of cumulative dose, must be estimated. The aims of the present study were (i) to propose a workflow for designing a MidP‐based PTV using both cardiac‐gated 4D‐CT and respiration correlated 4D‐CT data, and (ii) to evaluate the potential dosimetric benefits, in terms of cumulative dose, of the MidP approach in comparison with the ITV‐based strategy, for treatment planning of stereotactic radioablation of VT.

## 2 MATERIALS AND METHODS

### 2.1 Patients and treatment delivery

Data from 15 patients who underwent a STAR for refractory VT in our institution between October 2020 and January 2025 were included in this retrospective planning study. All patients were treated on a VersaHD linear accelerator (Elekta, Crawley, UK) using a volumetric modulated arc therapy (VMAT) technique using a cone‐beam CT (CBCT) image guidance procedure. The treatment was delivered using 2 coplanar VMAT arcs of around 270°. Before each arc, a first CBCT scan was acquired for patient repositioning, achieved through registration with the average respiratory CT scan, followed by manual adjustments. A second CBCT scan was performed after couch correction to verify the accuracy of the repositioning. Target localization was validated jointly by a radiation oncologist and a cardiologist before each arc delivery. One last CBCT scan was acquired after the second arc. During the whole treatment delivery, the position of the patient was monitored using a Catalyst surface‐guided system (C‐RAD, Stockholm, Sweden). A radiation dose of either 20 or 25 Gy, depending on the localization of the target and OARs, was delivered in a single treatment session, using two arcs. All patients were treated in a head‐first supine position on a comfortable mattress using an arm and knee support.

### 2.2 Cardiac and respiratory 4D‐CT imaging

Each of the 15 patients had an electrocardiogram (ECG) gated four‐dimensional computed tomography (4D‐CT_card_) scan and a respiratory‐gated 4D‐CT scan (4D‐CT_resp_). The 4D‐CT_card_ was acquired, during breath‐holding at submaximal inspiration on a Somaton Force 384 slice CT scanner (Siemens, Munich, Germany). To visualize the cardiac cavities, intravenous contrast was administered. Ten CT images, indexed on 10 cardiac phases, were retrospectively reconstructed based on the ECG signal recorded during scan acquisition. Additionally, an average cardiac CT dataset was reconstructed from the 10 phases.

The 4D‐CT_resp_ was acquired under free breathing on a Confidence 24‐slice CT scanner (Siemens, Munich, Germany). A Sentinel surface‐guided system (C‐RAD, Stockholm, Sweden) was used to record respiratory signal during image acquisition and for image binning. Ten respiratory phase‐binned CT images were retrospectively reconstructed. An average respiratory CT was also reconstructed from the 10 phases of the respiratory CT scans.

### 2.3 CTV delineation and assessment of cardiac and respiratory CTV motion

A cardiologist manually performed CTV delineation for each patient in the end‐of‐diastole phase of the 4D‐CT_card_, focusing on the cardiac region responsible for arrhythmia. To identify the arrhythmogenic region, other imaging modalities such as the electro‐anatomical map (EAM) acquired during previous cardiac radiofrequency ablation and/or PET‐CT were used.^17^

The motions of the target resulting from cardiac and respiratory dynamics were then estimated from 4D‐CT_card_ and 4D‐CT_resp_, respectively, using a previously published approach.^7^ Briefly, to estimate the target motion related to heartbeats, the CTV was propagated to the nine other cardiac phases using diffeomorphic demons deformable image registration (DIR).^18^ All propagated CTV were then associated to the average cardiac CT scan. Respiratory motions were estimated using rigid registrations of the average cardiac CT scan with each phase of the 4D‐CT_resp_. the 10 cardiac CTV to the 10 phases of the 4D‐CT_resp_. Throughout the entire process, the registration results underwent visual inspection and validation by both a radiation oncologist and a cardiologist.

For each patient, cardiac and respiratory motion amplitudes were assessed in the left/right (LR), antero/posterior (AP), and cranio‐caudal (CC) directions. Cardiac motion amplitude was defined in each direction as the difference between the maximum and minimum values of the CTV centroid DICOM coordinates in the ten cardiac phases of the 4D‐CT_card_. In the same manner, the respiratory motion amplitude was computed considering the CTV centroid in the ten respiratory phases of the 4D‐CT_resp_.

### 2.4 Localization of the mean time‐weighted cardio‐respiratory position of the CTV

The MidP approach is based on two main steps: (i) the identification of the mean time‐weighted position of the CTV, which is referred to as the MidP CTV (CTV_MidP_); (ii) application of PTV margins around this CTV_MidP_ to account for systematic and random errors.

**FIGURE 1:**
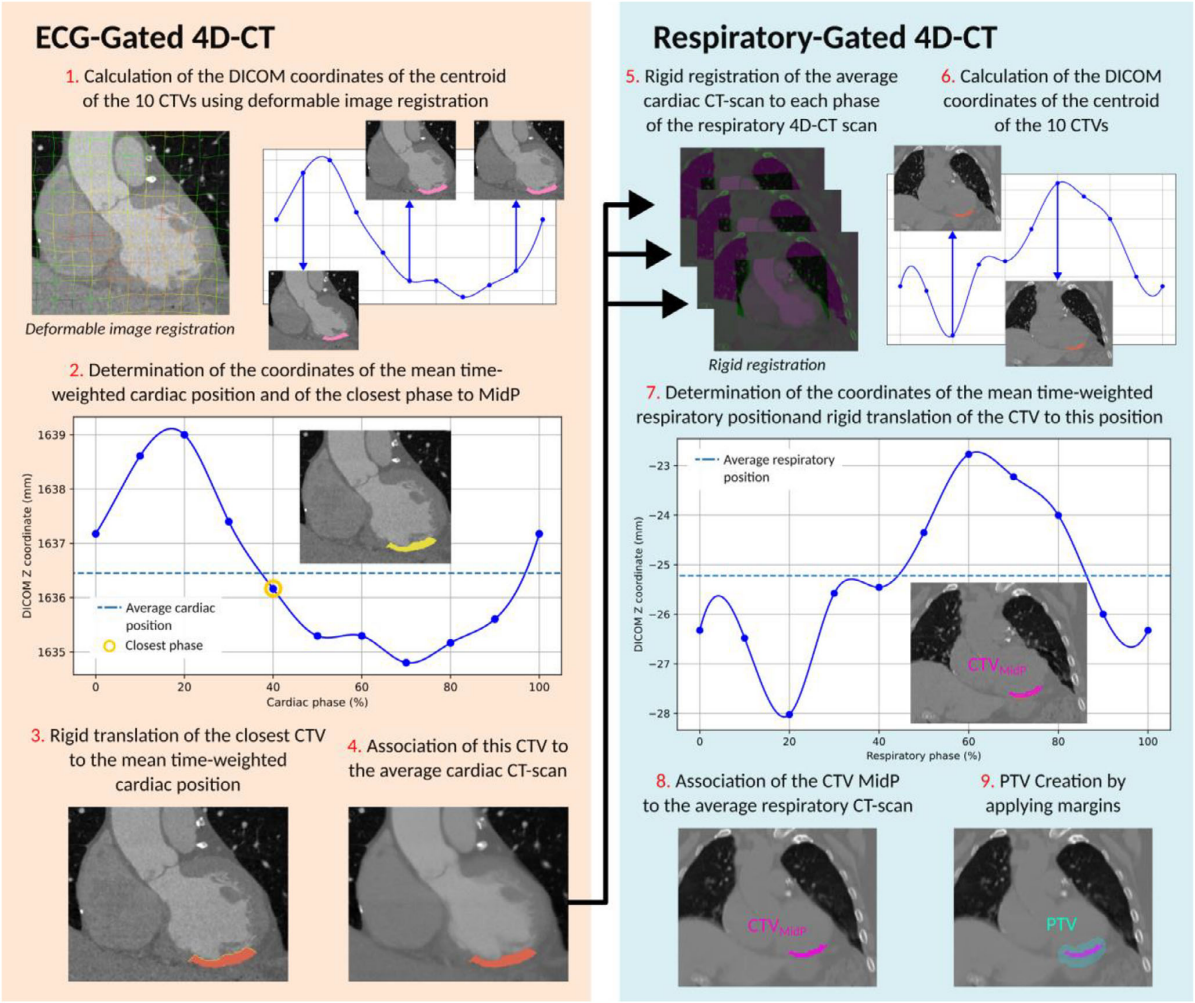
Workflow of identification of the mean time‐weighted cardiorespiratory position of the CTV. From the ECG‐gated 4D‐CT, the mean time‐weighted cardiac CTV was generated by translating the nearest CTV to the cardiac mean position and associated to the cardiac averaged CT scan. From the rigid registration of the cardiac average CT scan on each respiratory phase of the respiratory‐gated 4D‐CT, the respiratory MidP coordinates were determined. The CTV_MidP_ was created by translating the mean cardiac CTV to respiratory MidP coordinates. The PTV was then created by adding margins to the CTV_MidP_ after associating it to the respiratory average CT scan. 4D‐CT, four‐dimensional computed‐tomography; CTV, clinical target volume; CTV_MidP_, MidP CTV; ECG, electrocardiogram; MidP, mid‐position; PTV, planning target volume.

The CTV_MidP_ was extracted from both cardiac 4D‐CT data and respiratory 4D‐CT data (Figure 1). First, from the 10 cardiac phases of the 4D‐CT_card_ image set, DICOM coordinates of the mean time‐weighted cardiac position of the CTV centroid were calculated. Then, the closest CTV was rigidly translated to this mean position and then associated with the average cardiac CT‐scan. It was transferred to each phase of the respiratory CT scan using the result of the rigid registrations. DICOM coordinates (LR, AP, and CC) of the mean time‐weighted position of the 10 propagated CTV were finally calculated. The closest CTV was rigidly translated to this position, resulting in the final CTV_MidP_. This CTV_MidP_ was associated with the average respiratory CT‐scan.

### 2.5 MidP based PTV margins

MidP‐based PTV margins were calculated using the Van Herk recipe for CTV to PTV margins.^13^ (Equation 1):

(1) $$M_{\mathrm{PTV},\mathrm{MidP}}=a\Sigma +\beta \left(\sigma -\sigma_{P}\right)$$

where Σ is the standard deviation of systematic uncertainties, σ is the standard deviation of random uncertainties, σ_p_ is the parameter of the Gaussian defining the penumbral width, α is a parameter related to the confidence level for the CTV coverage and β is a parameter that depends on the prescription isodose level chosen to surround the PTV^13^ α is a parameter related to the confidence level for the CTV coverage and β is a parameter that depends on the prescription isodose level chosen to surround the PTV.^13^ In this study, an α value of 2.5 was chosen, ensuring that 90% of patients in the population receive a minimum cumulative CTV dose of at least the dose prescribed to the PTV. β was set to 0.84 as the prescription isodose was 80% of the maximum dose for the patient cohort.^13^ This approach excludes rotational and CTV deformation errors.^13^

When considering the different sources of errors occurring in the context of STAR, Equation (1) was decomposed in Equation (2):

(2) $$\begin{array}{ccc} M_{\mathrm{PTVMidP}} & & =2.5\Sigma_{\mathrm{eff}}+0.84\\ & & \;\left(\sqrt{\sigma_{\mathrm{eff}}^{2}+\sigma_{\mathrm{card}}^{2}+\sigma_{\mathrm{resp}}^{2}+\sigma_{p}^{2}}-\sigma_{P}\right) \end{array}$$

with:

(3) $$\sum_{\mathrm{eff}}=\sqrt{\sum_{\mathrm{intra}-\mathrm{fraction}}^{2}+\sum_{\mathrm{iso}}^{2}+\frac{1}{N}\left(\sigma_{\mathrm{intra}-\mathrm{fraction}}^{2}+\sigma_{\mathrm{voxel}}^{2}\right)}$$

(4) $$\sigma_{\mathrm{eff}}=\sqrt{\left(1-\frac{1}{N}\right)\left(\sigma_{\mathrm{intra}-\mathrm{fraction}}^{2}+\sigma_{\mathrm{voxel}}^{2}\right)}$$

where σ_card_ and σ_resp_ represent the errors associated with cardiac and respiratory motions, respectively, Σ_intra‐fraction_ and σ_intra‐fraction_ are errors related to intra‐fraction patient motion, σ_voxel_ describes the uncertainty due to the finite size of voxel in images, Σ_iso_ is the imaging‐to‐treatment isocenter misalignment, and *N* is the number of fractions of the treatment. The methods to estimate these errors are described below.

The van Herk margin recipe reported in Equation (1) has been devised for fractionated treatments. Several authors^19^, ^20^, ^21^ have noted that, when applying this method to an hypofractionated treatment regimen, the computation of random and systematic errors should consider the number of fractions *N*, resulting in Equations (3) and (4). As the single treatment session was delivered using two arcs and that the patient was re‐positioned, using a CBCT image guidance procedure before each arc, a value of *N* = 2 was used. To study the influence of this parameter, the margins were also computed using *N* = 4. In this study, no uncertainty due to target delineation was included. Among all components, only those associated with cardio‐respiratory motions were individually calculated for each patient, while the others were derived from the patient cohort or from standard values.

#### 2.5.1 Cardiac and respiratory motions induced errors

σ_card_ and σ_resp_ have been calculated individually for each patient based on the cardiac and respiratory 4D‐CT study. For respiratory motions, Van Herk et al. have shown that the associated error σ_resp_ was defined as 0.358 multiplied by the respiratory motion amplitude.^22^ No value was found in the literature for the cardiac random error; we make the assumption that the formula of the respiratory error proposed by Van Herk et al.^13^ kept a good estimator of the cardiac random error σ_card_.

#### 2.5.2 Intra‐fraction patient motion induced errors

Σ_intra‐fraction_ and σ_intra‐fraction_ are related to motions occurring during the treatment.^23^ They were quantified from the analysis of the CBCT images acquired before and after each arc delivery for the 15 patients.

Patient motion during an arc was assessed by performing rigid registrations between the CBCT acquired just after and the CBCT acquired just before the arc delivery. All registrations were performed on MIM 7.0.4 (MIM Software, Cleveland, OH). Σ_intra‐fraction_ was calculated by determining the standard deviation of the average intra‐fraction motion between arcs per patient and σ_intra‐fraction_ was obtained as the root mean square of individual deviations. Considering that there were only two arcs, it was decided to use the range of the intra‐fraction motions evaluated for each arc instead of the standard deviation for representing these individual deviations.

#### 2.5.3 Additional errors

Since the target (part of the heart) is mainly surrounded by soft tissue, the standard value for σ_p_ in water (i.e. 3.2 mm) was used^13^ Σ_iso_ is the residual imaging to treatment isocenter misalignment of the treatment unit. Its value was based on the analysis of periodic QA tests, as described in ref. [10]. We made the assumption that the finite size of the voxels of the CBCT and CT images induced a spatial uncertainty (σ_voxel_) for patient repositioning that can be estimated from a rectangular a priori probability distribution with a width equal to the voxel size^24^ For this rectangular probability distribution, the related standard deviation was estimated by: σ_voxel_ = voxel size / √12.

### 2.6 ITV‐based PTV generation

For each patient, an ITV‐based PTV (PTV_ITV_) was also generated. PTV_ITV_ was designed by using the approach clinically used in our institution and previously described.^7^ A cardiac ITV was first generated by taking the union of the 10 CTVs on the 4D‐CT_card_. This cardiac ITV was associated to the cardiac average CT‐scan and then propagated to each phase of the 4D‐CT_resp_ using the transformation obtained by rigid registration. The cardio‐respiratory ITV was created as the union of the 10 propagated cardiac ITVs. Finally, the PTV_ITV_ was created by adding an ITV‐to‐PTV_ITV_ margin of 3 mm addressing mostly intra‐fraction patient motion uncertainties.

### 2.7 Treatment planning

For both PTV_MidP_ and PTV_ITV_, a treatment plan was optimized on RayStation Treatment Planning System (TPS) (RaySearch Laboratories AB, Stockholm, Sweden) using the planning protocol used in our institution for STAR. All treatments plans were generated using a VMAT technique on a VersaHD linac. The VMAT treatment plans were designed using two coplanar arcs of 270° with a 6 MV flattening filter‐free (FFF) photon beam. A dose of 25 or 20 Gy was prescribed to the PTV in a single fraction. A prescription of 20 Gy was chosen for patients for which the CTV was close to the left coronary arteries or close to the stomach. The dose was prescribed to the 95% PTV coverage for all treatment plans (i.e., PTV D95% = 25 Gy (or 20 Gy)). The maximum dose allowed within the PTV was 125% (± 3%) of the prescribed dose. The treatments plans were optimized to respect the organs at risk dose constraints from the RAVENTA trial.^25^ OARs were delineated on the average respiratory CT‐scan without addition of any margins taking the assumption that the average CT scan implicitly included (cardio)respiratory motions. To enhance the dose fall‐off, tuning structures, including 3‐mm and 20‐mm rings, were used.

### 2.8 Data analysis

#### 2.8.1 PTV volumes

For the patient cohort, the volumes of PTV_MidP_ were compared to the volumes of PTV_ITV_. The PTV_MidP_ for each individual patient were generated without including the intra‐fraction displacement measured for this patient in the population parameters, following a “leave‐one‐out” scheme.

For comparison purpose, PTVs were also generated with *N* = 4, while reusing the same values for the other parameters.

#### 2.8.2 4D dose accumulation

For each treatment plan and PTV, we evaluated the accumulated dose to the CTV and organs at risks using a dynamic motion model developed previously by our group.^26^ Studied OARs included the heart, esophagus, stomach, coronary arteries, aorta, lungs, left atrium, and superior vena cava. They were all segmented using the TotalSegmentator^27^ algorithm.

For the CTV and intra‐cardiac OARs (left coronary artery and left atrium), the corresponding structure was delineated on one phase of the cardiac CT scan, then the corresponding dynamic model was generated based on its cardio‐respiratory motions. These motions were estimated from deformable registration of the phases of the cardiac 4D‐CT, then rigid registrations of the average cardiac 4D‐CT to each phase of the respiratory 4D‐CT, as described in section I.C.

Extra‐cardiac OARs were delineated on one phase of the respiratory 4D‐CT. Corresponding dynamic models were computed by estimating respiratory motions of each structure using deformable registration of the phases of the respiratory 4D‐CT with the diffeomorphic demons algorithm.

Intra‐fraction motions (Section 2.5.2) were finally incorporated for each arc to the motion models during treatment simulations. The position of each structure was tracked and interpolated at 30 ms time steps during a 5 min duration corresponding to a realistic irradiation time in STAR. At each time step, the corresponding dose value for each point was extracted from the dose matrix. The cumulative dose was computed by averaging these values for each voxel of the CTV. Dose indices relative to CTV and OARs irradiation were calculated from this data.

#### 2.8.3 Statistical analysis

A Wilcoxon signed‐rank test was used to calculate the *p*‐value to evaluate the statistical significance of the results. The results were considered as statistically significant if the *p*‐value was lower than or equal to 0.05.

## 3 RESULTS

### 3.1 Cardio‐respiratory motion amplitude and MidP margins

**FIGURE 2:**
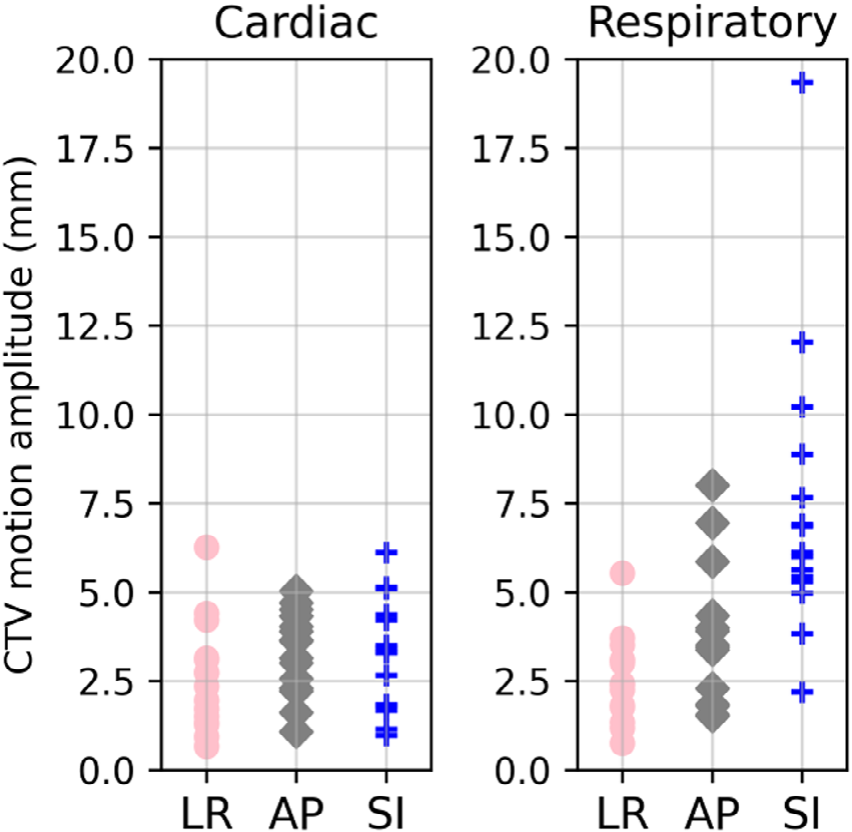
Target motion amplitudes for the 15 patients. Target cardio‐respiratory motion amplitudes in the 3 directions obtained from the study of the cohort of 15 patients.

The cardiac and respiratory motion amplitudes of the CTV in LR, AP, and CC directions are reported in Figure 2 for the 15 patients. Cardiac motions had a mean [min, max] value of 2.5 [0.7, 6.3] mm, 3.4 [1.1, 5.0] mm, and 3.0 [1.0, 5.2] mm in the LR, AP, and CC directions, respectively. Respiratory motions had a mean value of 2.6 [0.7, 6.3] mm, 3.5 [1.5, 7.0] mm, and 7.0 [2.2, 12.0] mm in the LR, AP, and CC direction, respectively.

Systematic and random error values of ∑_iso_, ∑_intra‐fraction_, σ_voxel_, and σ_intra‐fraction_, calculated from the patient cohort, are reported in Table 1, with the resulting PTV margins. The MidP‐based strategy led to margins of 4.2 [4.0, 4.7] mm, 4.0 [3.7, 4.4] mm, and 4.9 [4.2, 5.9] mm on average in the LR, AP, and CC directions, respectively. For each patient and in these three directions, the margins computed with the leave‐one‐out strategy differed by an average of 0.1, 0.1, and 0.2 mm from the ones computed with the complete patient data.

**TABLE 1:** Systematic and random errors of the patient group for the MidP strategy, and calculated margins.

	LR	AP	CC
Σ_intra‐fraction_ (mm)	0.8	0.8	1.0
σ_intra‐fraction_ (mm)	1.7	1.4	1.6
Σ_iso_ (mm)	0.4	0.3	0.3
σ _voxel_ (mm)	0.6	0.6	0.6
σ_card_ and σ_resp_	Patient‐specific		
M_PTV Midp_ (mean, [min, max]) (mm)	4.2, [4.0, 4.7]	4.0, [3.7, 4.4]	4.9, [4.2, 5.9]

*Note*: The errors associated with cardio‐respiratory motions are not included, as they are calculated individually for each patient.

Comparatively, the margins obtained using *N* = 4 were of 3.5 [3.2, 4.1] mm, 3.5 [2.9, 3.8] mm, and 4.4 [3.6, 5.4] mm on average in the LR, AP, and CC directions, respectively.

### 3.2 Planning target volumes

The patients presented CTVs in various locations in the LV myocardium. The most represented CTV location was basal‐anterior and basal‐anteroseptal. The CTVs were delineated with an average volume of 25.6 [16.6, 45.5] cc.

**FIGURE 3:**
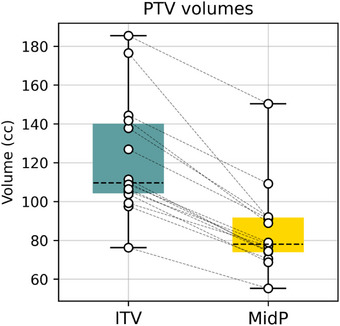
PTV for the different approaches. Each point represents an individual patient. PTV, planning target volume.

The volumes of MidP‐based PTV and ITV‐based PTV are reported in Figure 3. Compared to the ITV‐based method, the MidP‐based strategy resulted in a reduction of PTV for all patients of the cohort (*p* < 0.001) with a mean [min, max] PTV reduction of 30 [19, 42] %.

Comparatively, the PTVs obtained using *N* = 4 presented a reduction of 36 [25, 49] % compared to the ITV‐derived PTVs.

### 3.3 Dosimetric evaluation

#### 3.3.1 CTV coverage

**FIGURE 4:**
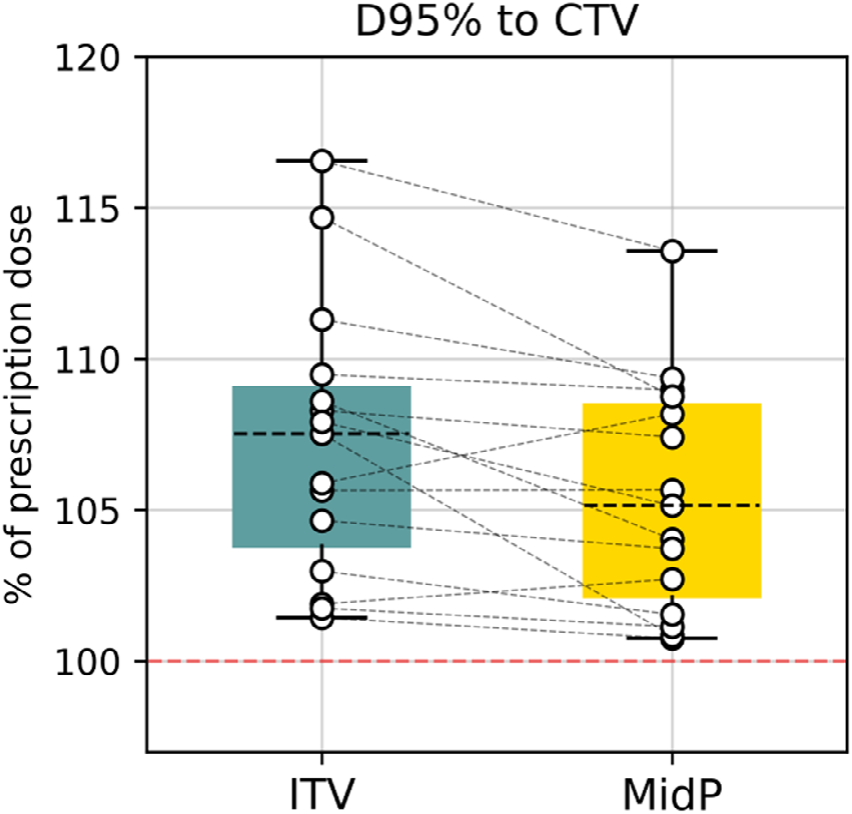
Dose received by 95% of the CTV for ITV and MidP approaches. The dashed red line marks the level of 100% of the prescribed dose delivered to 95% of the CTV. CTV, clinical target volume; ITV, internal target volume; MidP, mid‐position.

Figure 4 illustrates the dose covering 95% of the CTV for both strategies. While a significant reduction was observed in D95% using MidP (*p* = 0.010), the D95% remained superior to the prescribed dose for all patients. To facilitate comparison, the values were normalized to the prescribed dose, which varied among patients (20 or 25 Gy).

Table 2 shows main dose indices for the CTV using ITV and MidP strategies, as well as the corresponding *p*‐values. Among all reported dose indices, a significant difference (*p*‐value < 0.05) was found for all indicators but D_98%_ between MidP and ITV strategies with lower indices values for the MidP strategy. No statistical difference was found for D_100%_ and for the indices relative to the proportion of the target covered by the prescribed dose (V_X%_).

**TABLE 2:** Comparison of DVH parameters (mean, [min, max]) between the two strategies for the accumulated 4D dose to the CTV.

	ITV	MidP	*p*‐value
D_mean_ (%)	114, [107, 124]	112, [106, 119]	0.0009
D_50%_ (%)	114, [106, 125]	112, [105, 119]	0.0059
D_2%_ (%)	122, [116, 131]	120, [114, 127]	0.0256
D_95%_ (%)	107, [101, 117]	105, [101, 114]	0.0103
D_98%_ (%)	106, [101, 115]	104, [99, 112]	0.0554
D_99%_ (%)	105, [101, 113]	104, [99, 111]	0.0479
D_100%_ (%)	101, [93, 106]	97, [86, 103]	0.0006
V_100%_ (%)	100, [99.5, 100]	100, [96, 100]	0.2357
V_98%_ (%)	100, [100, 100]	100, [99, 100]	0.0679
V_95%_ (%)	100, [100, 100]	100, [100, 100]	0.1088

*Note*: All values are normalized to the prescribed dose.

#### 3.3.2 Dose to OAR

**FIGURE 5:**
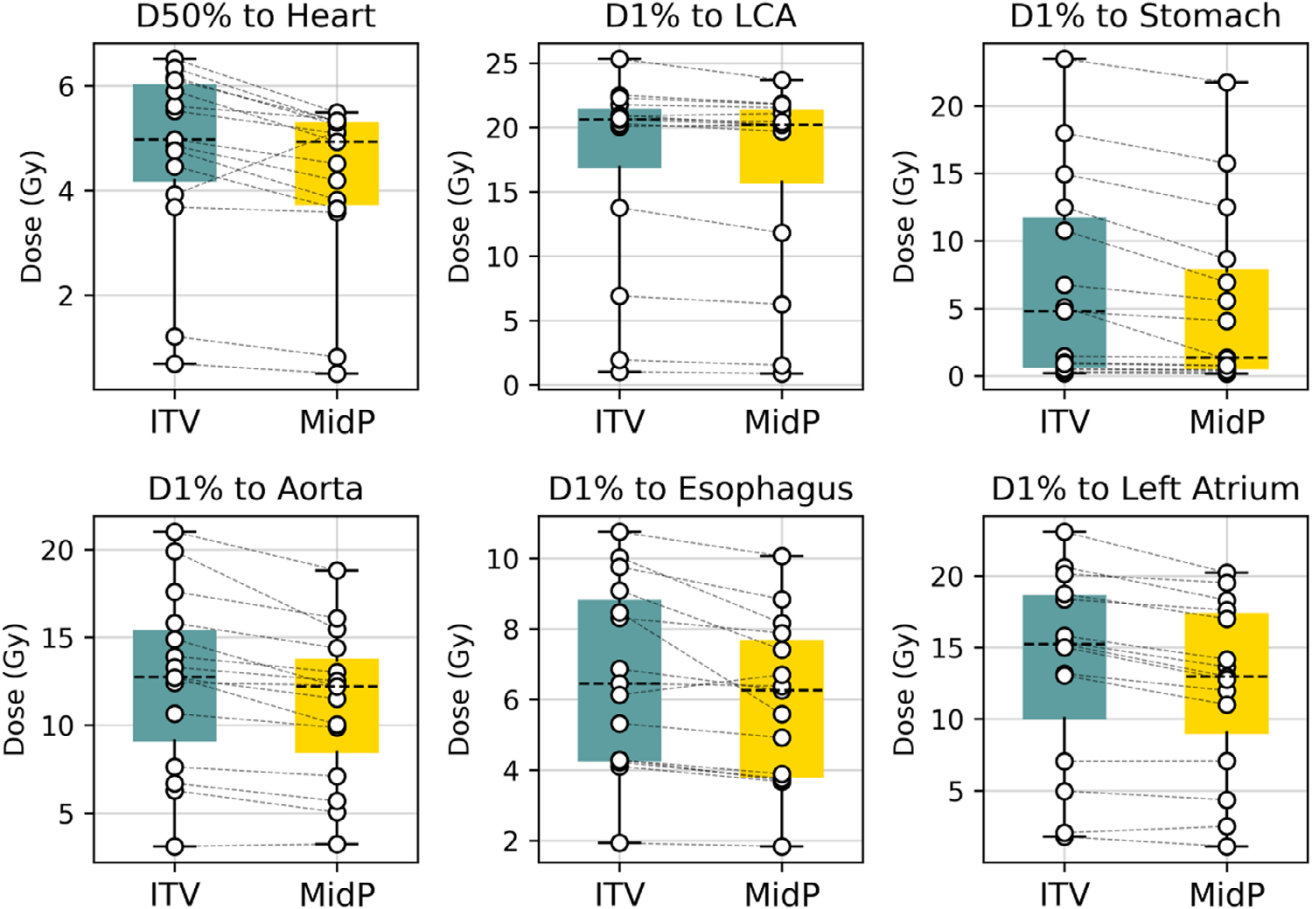
Dose indicators for multiple organs at risk.

Table 3 provides, for each strategy, the dose indices for the main organs at risk. The indices are also reported in Figure 5 for the left coronary artery, stomach, left atrium, esophagus, aorta, and whole heart. The MidP strategy provided improvements for all organs, with significant values for all of them.

**TABLE 3:** Comparison of dose to organs at risk (mean, [min, max]) between the three strategies for the planned dose.

Organ at risk	DVH parameter	ITV	MidP	*p*‐value
Whole heart	D50% (Gy)	4.7, [0.7, 6.5]	4.2, [0.5, 5.5]	0.0084
Coronary arteries	D1% (Gy)	17.3, [1.0, 25.3]	16.8, [0.9, 23.7]	0.0012
Stomach	D1% (Gy)	6.7, [0.2, 23.5]	5.4, [0.2, 21.8]	0.0007
Aorta	D1% (Gy)	12.6, [3.1, 21.0]	11.2, [3.2, 18.8]	0.0008
Esophagus	D1% (Gy)	6.7, [1.9, 10.8]	5.9, [1.8, 10.1]	0.0015
Lungs	D5% (Gy) D50% (Gy)	10.5, [3.2, 21.0] 1.0, [0.2, 2.6]	9.2, [2.7, 19.0] 0.8, [0.1, 1.6]	0.0007 0.0008
Skin	D1% (Gy)	4.9, [2.2, 7.3]	4.4, [1.9, 6.2]	0.0007
Left Atrium	D1% (Gy)	13.6, [1.8, 23.1]	12.3, [1.1, 20.2]	0.0003
Superior Vena Cava	D50% (Gy)	1.1, [0.0, 2.7]	0.9, [0.0, 2.4]	0.0063

The median dose to the whole heart (D50%), which is the most common heart dose constraint used for STAR treatment planning^25^ was significantly lower with the MidP strategy (*p* = 0.008) with a mean reduction of 0.5 Gy.

For the lungs, both D5% and D50% showed significant decreases with MidP, with average reductions of 1.3 and 0.2 Gy, respectively.

**FIGURE 6:**
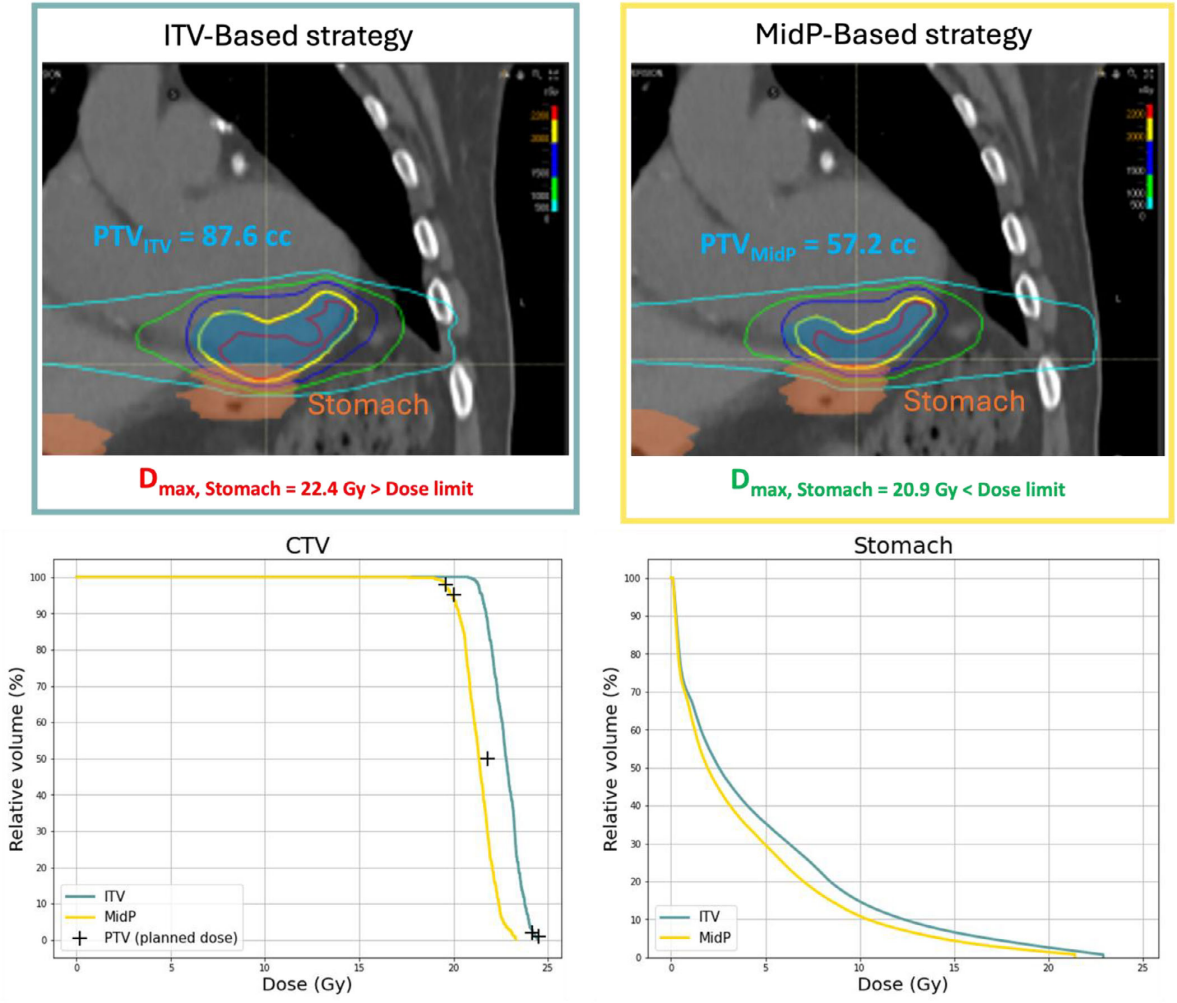
Comparison of ITV and MidP strategies for a patient with target volume close to the stomach. Top: PTV and planned dose distributions obtained for the ITV‐based and MidP‐based strategies. Bottom: DVH showing the accumulated dose to the CTV and the stomach for the two strategies for the same patient. For the CTV, D98%, D95%, D50%, D2%, and D1% of the planned dose to the PTV are also outlined. CTV, clinical target volume; ITV, internal target volume; MidP, mid‐position; PTV, planning target volume.

MidP also resulted in a significant reduction of 19% in the D1% for the stomach (*p* < 0.001). For one patient, the ITV‐based strategy resulted in a maximum dose to the stomach of 22.4 Gy, which was decreased to 20.9 Gy for the MidP strategy, thus allowing to meet the dose constraint (< 22 Gy) for this organ at risk^8^ For this patient, the planned dose distributions in the 3 directions are presented in Figure 6 along with the DVH showing the accumulated dose to the CTV and the planned dose to the stomach.

Differences in near maximum doses (D1%) to coronary arteries, left atrium and aorta were all statistically significant. Notably, one patient had a significant reduction in the maximum dose to the aorta, decreasing from 19.9 to 15.5 Gy using the MidP strategy.

## 4 DISCUSSION

STAR is still a recent and experimental strategy for treating patients with refractory VT. Its implementation is a multidisciplinary challenge, in which several uncertainties may appear throughout the process. The treatment itself is delivered with a high radiation dose directed to the myocardium of the patient. Therefore, the patient‐specific or treatment‐related uncertainties must be accounted for to ensure a good coverage of the target. This is often done through addition of conservative margins to the treatment volumes, that can then become very large and threaten the safety of the procedure. In this work, we compared planning treatment volumes generated through a conservative ITV method to planning target volumes generated by using the MidP formalism, that was meant to reduce the overall irradiated volume while keeping a good CTV coverage. To our knowledge, the present work is the first study which describes the application of the MidP strategy to incorporate cardio‐respiratory motions and treatment uncertainties into the PTV for STAR, and evaluates it in terms of volume reduction, target coverage and dose to organs at risk in comparison to the more commonly used ITV‐based strategy.

Firstly, geometrical comparisons of the volumes generated using both methods showed a systematic reduction of PTV volumes (30%, [19%, 48%]) using the MidP strategy over the ITV. This PTV reduction is in agreement with the volume reduction trends found in the literature for other applications (reductions of 23%, 25% and 14% in PTV size have been reported for the MidP strategy compared to the ITV strategy for the treatment of pulmonary and pancreatic cancerous lesions^11^, ^14^, ^16^). For comparison purpose, we also generated PTVs with the MidP process using parameter N = 4, which represents a hypothetical treatment with three intermediate steps of patient repositioning. This led to smaller PTVs compared to MidPosition PTV considering 2 subfractions (N = 2). However, this increased complexity in the workflow may compromise patient comfort during treatment delivery, potentially leading to greater intra‐fraction displacements and thereby negating any benefits.

The margins obtained using the leave‐one‐out scheme showed minimal differences from those obtained using the complete dataset. This shows that, despite the limited cohort of 15 patients, the computed margins converge to values that are adequate for all patients.

Reductions of target volumes observed using the MidP workflow are expected to come along with a reduction of the radiation dose received by the treatment target and by surrounding organs. In this work, the dose to CTV and OAR with both ITV and MidP methods was evaluated using a 4D dose accumulation process including all the cardio‐respiratory motions as well as the intra‐fraction patient motions occurring during STAR treatments.

This analysis showed that the MidP strategy, compared to an ITV approach, allowed a consistent reduction in dose delivered to OARs surrounding the CTV, including the heart, coronary arteries, the stomach, the aorta, the left atrium and the lungs. In the context of STAR for VT, the patients usually have a fragile health condition. Moreover, a grade 4 toxicity related to stomach^28^ and a lethal esophago‐pericardial fistula^29^ were reported in the literature for STAR treatment. It is thus of crucial importance to limit the radiation dose received by organs surrounding the CTV.

While these reductions in dose to OARs are welcome to increase patient safety, adequate target coverage must be ensured. There were no significant differences in the volume of CTV receiving the prescribed dose between the two strategies. The experiments showed an average reduction in target coverage for the considered patients, that remained adequate for treatment efficacy, with the D95% exceeding 100 % of the prescribed dose for each patient. It should be noted that the definition of acceptable CTV coverage in STAR treatment is not well‐established in the existing literature, thus we considered values commonly used for cancer treatment. Knutson et al.^8^ aimed for 95% of the PTV to receive 95% of the prescribed dose, however in our study the treatment plans were designed such as 95% of the PTV received the prescribed dose, which may impact the results.

It is important to emphasize that MidP strategy used the van Herk PTV margin recipe which has been developed for fractionated treatments, and not for single‐session treatment schemes. Although the 4D cumulative dose approach implemented in our study shows that, for our patient cohort, the CTV to PTV margins derived from the van Herk formula provided adequate CTV coverage, its clinical implementation for STAR remains a proposed strategy that still requires validation. Further studies focusing on plan robustness are warranted to more fundamentally evaluate the applicability of the van Herk formula for single‐session treatments, especially in scenarios involving small patient cohorts, such as those encountered in STAR. In particular, it would be valuable to investigate, in a more robust manner, the impact of the number of intermediate patient positioning corrections (i.e., the number of subfractions) on the definition of PTV margins for these single‐session treatments. In this context, it is essential to conduct an initial, institution‐specific validation of PTV margins before implementing the MidP strategy in clinical practice to ensure its appropriate application. This validation should include a retrospective, patient‐specific assessment of CTV coverage that accounts for individual motion patterns, for example, through a 4D dose accumulation approach, such as the one proposed in this study.

This study has some limitations. Firstly, it is assumed that dividing the treatment into two sub‐fractions with an intermediate repositioning step allows it to be considered as two separate fractions in the margin calculation. Additionally, the dynamic model used to calculate the accumulated dose to the CTV was based on a convolution of the static dose map generated from the TPS with the CTV motion rather than a dosimetric re‐calculation of the dose in each cardiac and respiratory phase.^26^ Thus, it did not consider the dose deposition associated with the specific position of the patient's anatomy at a given moment in time, especially at the heart/lung interfaces. Then, to compute MidP margins, the width of the penumbra was considered in 3 directions as the value proposed by Van Herk et al. in water. An individualization in function of the target position could be performed by using the value associated with air when the target is against the lung. However, this method was tested for a few patients of this study, and it showed that penumbra had very little influence on the final margin. Finally, the experiments were conducted using the data of a limited number of 15 patients. This can be partially explained by the fact that STAR is a relatively new treatment, for which few patients are enrolled.

In terms of ease of implementation, the ITV approach is the simplest to use. On the other hand, the MidP strategy requires an assessment of various data from a patient cohort to generate the margins. It means that a substantial number of patients who have already undergone treatment is needed to use their data retrospectively. Additionally, the MidP strategy requires the selection of the CTV MidP, the delineation of the CTV on the cardiac CT scan and the evaluation of its cardiac motions necessitates advanced tools for deformable registrations, which are however becoming more and more integrated into commercial solutions.

## 5 CONCLUSION

In this work, we proposed a method based on the use of cardiac and respiratory 4D‐CT imaging data to implement the MidP concept for designing the planning target volume for STAR. The mid position strategy was evaluated using data of 15 patients and was compared to a more classical ITV‐based strategy using geometric and dosimetric criteria.

Compared with the ITV approach, the use of the MidP strategy for STAR lead to a significant reduction of planning target volumes, while still maintaining an acceptable clinical target volume coverage. The MidP strategy improves dose sparing of OAR including the heart and is particularly advantageous for targets located close to the diaphragm for which the stomach is a major concern. This study suggests that the MidP strategy should be considered to optimize the delivery of STAR when no active cardio‐respiratory motion management technique is available.

## AUTHOR CONTRIBUTIONS

Adèle Gabillaud, Louis Rigal, Raphaël Martins, Renaud de Crevoisier, Loïg Duvergé, Nolwenn Delaby, Karim Benali, Julien Bellec, and Antoine Simon contributed to the conception and design of the study. Adèle Gabillaud, Louis Rigal, Juan Cisneros, Julien Bellec, and Antoine Simon performed experiments, evaluation, and analysis. Mathieu Lederlin provided clinical imaging data. Adèle Gabillaud wrote the first draft of the manuscript. All authors contributed to the manuscript revision, read and approved the submitted version.

## CONFLICT OF INTEREST STATEMENT

The authors declare no conflicts of interest.
